# Genetic analysis in the clinical management of biliary tract cancer

**DOI:** 10.1002/ags3.12334

**Published:** 2020-04-14

**Authors:** Toshifumi Wakai, Masayuki Nagahashi, Yoshifumi Shimada, Pankaj Prasoon, Jun Sakata

**Affiliations:** ^1^ Division of Digestive and General Surgery Niigata University Graduate School of Medical and Dental Sciences Niigata Japan

**Keywords:** biliary tract cancer, cholangiocarcinoma, genetic analysis, genome medicine, surgical oncology

## Abstract

Biliary tract cancer (BTC) is clinically and pathologically heterogeneous and responds inadequately to treatment. A small section of patients develop resectable disease, although the relapse rates are high; the benefits of adjuvant capecitabine chemotherapy for BTC are now understood, and gemcitabine‐based combination chemotherapy is the first line of therapeutic strategy for BTC; however, alternative therapy for BTC is not known. Genomic profiling can provide detailed information regarding the carcinogenesis, identification, and therapy for BTC. Currently, confirmed restorative targets for BTC are lacking. In this review, we aimed to analyze the preclinical and clinical implications of a spectrum of genomic alterations associated with new potentially remedial targets. We focused on eight draggable genes for BTC, which were described as having evidence of therapeutic impact (evidence level 2A‐3B) based on the clinical practice guidance for next‐generation sequencing in cancer diagnosis and treatment; these include ERBB2, NTRK1, RNF43, CDK6, CDKN2B, FGFR2, IDH1, and IDH2. Moreover, some of the BTC present microsatellite instability, hypermutation, and germline variants, which we also reviewed. Finally, we discussed the therapeutic options based on the next‐generation sequencing findings in BTC. Studies have demonstrated that BTC includes subgroups with individually distinct driver mutations, most of which will be targeted with new treatment plans.

## Graphical abstract

Genomic profiling can provide detailed information regarding the carcinogenesis, identification, and therapy for biliary tract cancer. In this review, we aimed to analyze the preclinical and clinical implications of a spectrum of genomic alterations associated with new potentially remedial targets. NTRK gene fusions contribute to the transcription of chimeric TRK proteins with constitutively triggered or excessive expression of kinase function, which contribute to oncogenic possibilities.

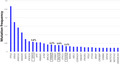

## 1 INTRODUCTION

Biliary tract cancers (BTCs) include intrahepatic cholangiocarcinoma, extrahepatic cholangiocarcinoma, and gallbladder carcinoma. BTC occurs in the epithelial cellular lining of the bile duct and may appear at specific anatomical regions (intrahepatic, extrahepatic, and gallbladder). Although BTC is predominant in East and South Asian countries and in certain regions of South America, the worldwide prevalence of BTC is increasing rapidly.^1^, ^2^ Studies show that BTC is not a single distinct disorder, but consists of several diseases with specific demographics, molecular features, and treatment options.

Intrahepatic cholangiocarcinoma is the second‐most typical major hepatic malignancy, with an increasing global incidence, especially in the European hemisphere; this is perhaps because of the elevated percentage of overweight individuals and incidence of hepatitis C infection.^3^, ^4^, ^5^, ^6^, ^7^, ^8^, ^9^ In Asia, intrahepatic cholangiocarcinoma is principally associated with parasitic infections.^3^, ^4^, ^5^, ^6^, ^7^, ^8^, ^9^ The majority of extrahepatic cholangiocarcinoma occurs within the hepatic duct bifurcation; in 30% of cases, the disease develops within the distal common bile duct and occurs with pain‐free jaundice.^7^ Gallbladder carcinoma is undoubtedly an unusual disease linked to cholecystitis, cholelithiasis, and obesity.^7^ Although surgical resection is the most accepted treatment method for treating these tumors, the tumors are inoperable for a significant proportion of patients.^7^, ^10^, ^11^, ^12^ For patients with unresectable advanced/recurrent BTC, gemcitabine‐based combination chemotherapy is the first line of therapeutic strategy, such as gemcitabine plus cisplatin,^13^, ^14^ gemcitabine plus S‐1,^15^ and gemcitabine/cisplatin/S‐1 combination chemotherapy.^16^, ^17^ The efficacy of gemcitabine‐based combination chemotherapy on survival is encouraging; the median overall survival following these therapies has been reported to range from 11.2 to 16.2 months.^13^, ^14^, ^15^, ^16^, ^17^ On the other hand, no scientific molecular markers for earlier medical diagnosis have been identified, and effective specific molecular therapies are unavailable; consequently, the 5‐year survival rate is extremely minimal (10% for cholangiocarcinoma and <5% for gallbladder carcinoma).^4^, ^9^, ^18^ Hence, understanding the molecular features of this disease might assist in developing targeted therapeutics.^19^, ^20^

Patients with tumors developing in the vicinity of bile ducts present with biliary obstruction because of regional infiltration in the biliary tract. A small section of patients can probably be identified to have very early disease, which can be resected surgically. For patients clinically determined to have advanced disease frequently showing nonspecific and non‐biliary obstructive symptoms, treatment plans are non‐curative and predominantly based on chemotherapy. Despite this unmet healthcare requirement, the genomic and transcriptomic landscape of this tumor type remains inadequately identified, primarily regarding the distinction of its three anatomical subtypes. Herein, we reviewed the genetic alterations in BTC, focusing on intrahepatic cholangiocarcinoma, extrahepatic cholangiocarcinoma, and gallbladder carcinoma, with the aim of investigating potential therapeutic possibilities.

## 2 GENETIC ALTERATIONS IN BILIARY TRACT CANCER

Advancements in molecular biology have resulted in the recognition of numerous gene irregularities. Gene panel testing and the efficient use of genomic mutation analysis via next‐generation sequencing (NGS) or related approaches competent at synchronized recognition of multiple genomic mutations are utilized for elucidating cancer‐associated genomic mutation(s) in specific individuals and for designing the most suitable customized treatment. An average test panel addresses gene history, which is considered to be beneficial for forecasting responses to medication and prognosis, resulting in conclusive medical diagnosis. These panels can concurrently display a large number of transcripts and offer an array of information that uncovers genomic variations, which include gene mutations, deletions, insertions, gene fusions, and duplications. In addition, the extent to which information is integrated in gene panel tests is controlled by the innovations in diagnostic and treatment strategies.^21^

In 2017, Valle et al^22^ reported the molecular genetics of BTC, and, in 2018, Sunami et al^21^ reported the clinical practice guidance for NGS for cancer diagnosis and treatment. Eight of the genes altered in BTCs reported by Valle et al^22^ were described as having evidence of therapeutic impact (evidence level 2A‐3B) in “biliary cancer” or “solid tumor” based on the clinical practice guidance for NGS in cancer diagnosis and treatment^21^; these include *ERBB2* amplification (evidence level 2A), *NTRK1* fusion (evidence level 2A), *RNF43* mutation (evidence level 3A), *CDK6* (evidence level 3B) and *CDKN2B* loss (evidence level 3B), *FGFR2* fusion (evidence level 3B), *IDH1*, and *IDH2* mutations (evidence level 3B), listed in Table 1.

**TABLE 1:** Draggable genes with therapeutic impact for biliary tract cancer partly cited from the clinical practice guidance for next‐generation sequencing in cancer diagnosis and treatment (Edition 1.0)^21^

Gene name	Types of gene alterations	Tumor type^a^	Mutation frequency^b^	Clinical significance^a^	Evidence level^a^	Agents	Reactivity
*ERBB2*	Amplification	Biliary cancer	GBC 9.8%‐19% ECC 11%‐17%	Response	2A	Trastuzumab/Pertuzumab	Sensitive
*NTRK1*	Fusion gene	Solid tumor	ICC 5.6%	Response	2A	Pan Trk inhibitor	Sensitive
*RNF43*	Mutation (loss of function)	Solid tumor	GBC 3.9% ICC 9.3%	Response	3A	LGK974 (Porcupine inhibitor)	Sensitive
*CDK6*	Amplification/Actionable mutation	Solid tumor	ICC 7%	Response	3B	Ribociclib	Sensitive
*CDKN2B*	Mutation (loss of function)	Solid tumor	GBC 5.9%‐19% ECC 17% ICC 5.6%‐25.9%	Response	3B	CDK4/6 inhibitor	Sensitive
*FGFR2*	Fusion gene	Biliary cancer	GBC 3% ICC 11%‐45%	Response	3B	PD173074 (FGFR inhibitor)	Sensitive
*IDH1*	Actionable mutation	Biliary cancer	GBC 1.5% ECC 0.7%‐4% ICC 4.9%‐36%	Response	3B	Dasatinib	Sensitive
*IDH2*	Actionable mutation	Biliary cancer	GBC 1.5% ECC 0.7%‐4% ICC 4.9%‐36%	Response	3B	Dasatinib	Sensitive

Abbreviations: ECC, extrahepatic cholangiocarcinoma; GBC, gallbladder carcinoma; ICC, intrahepatic cholangiocarcinoma.

^a^ Tumor type, clinical significance, and evidence level of the draggable genes with therapeutic impact were cited from the clinical practice guidance for next‐generation sequencing in cancer diagnosis and treatment (Edition 1.0).^21^

^b^ Mutation frequency of all genes but one was cited from a review article reported by Valle et al.^22^ Mutation frequency of NTRK1 was quoted from a study reported by Ross et al.^49^

To see the frequency of gene alterations in BTCs in Asian patients, we downloaded the mutation data of 310 Asian cases with BTC (239 Japanese cases and 71 Singaporean cases) from the International Cancer Genome Consortium data portal. Among the eight genes described above as having evidence of therapeutic impact in the clinical practice guidance for NGS for cancer diagnosis and treatment,^21^ the genes most frequently altered were *IDH1* (5.8%), *ERBB2* (4.2%), *RNF43* (3.9%), and *FGFR2* (3.2%) in Asian patients with BTC.

## 3 HUMAN EPIDERMAL GROWTH FACTOR RECEPTOR (*HER*) FAMILY AND *ERBB2* AMPLIFICATION

Evidence indicates that *HER2* might be used as a novel restorative target in patients with BTCs.^23^ The frequency of *HER2* amplification or overexpression is noted in roughly 4%‐28.6% of GBC,^19^, ^23^, ^24^, ^25^, ^26^, ^27^, ^28^ 4%‐11% of extrahepatic cholangiocarcinoma,^26^, ^27^, ^28^ and 0.6%‐5% of intrahepatic cholangiocarcinoma.^26^, ^27^, ^28^ Previous reports show that the principal site of occurrence of *HER2*‐positive BTC varies; the ratio of HER2 3+ in immunohistochemistry was at its maximum in GBCs, followed by that in extrahepatic cholangiocarcinoma.^27^, ^29^ In addition, recent genomic research has shown how mutational information is BTC ‐specific, as indicated by their principal sites of occurrence or etiological factors.^19^, ^30^, ^31^, ^32^ Studies show that BTC pathogenesis might be unique for these variables, and therefore, various therapeutic approaches might be required, which will depend on patient‐specific medical details, as well as the outcomes of inherited or molecular profiling.^23^ Studies (using immunohistochemistry) have not been able to illustrate the prognostic effect of *HER2* overexpression in patients with BTC because of comparatively smaller sample size and heterogeneity of individual attributes.^23^, ^33^, ^34^ Some other studies have indicated that *HER2* overexpression is associated with poor prognosis of patients without any targeted therapies, and that these patients might benefit from targeting the HER2 signaling pathway.^35^, ^36^

Trastuzumab is a monoclonal antibody targeting HER2. Certain earlier studies encourage the application of trastuzumab‐based combination chemotherapy owing to its anti‐tumor activity in patients with *HER2*‐positive BTC.^23^, ^37^, ^38^, ^39^ Substantial, randomized, and controlled studies of *HER2*‐targeted therapies have been advantageous for patients with *HER2*‐positive gastroesophageal adenocarcinoma and breast cancer, as *HER2* overexpression and amplification is more frequent in these cancers. On the other hand, case reports and series have established HER2 as an efficient therapeutic target in patients with gallbladder carcinoma.^37^, ^38^, ^39^ Javle et al^39^ reported that in gallbladder carcinoma patients with distant metastases, trastuzumab was related to partial response (n = 4), stable disease (n = 3), or complete response (n = 1), resulting in a 56% response rate, whereas patients with cholangiocarcinoma did not respond to trastuzumab therapy. The MyPathway basket trial incorporated seven patients with *HER2* amplification or overexpression in BTC who were treated with *HER2*‐targeted therapy (trastuzumab and pertuzumab); two patients showed partial response, while the other three patients had stable disease beyond 120 days.^40^ In addition, the SUMMIT trial using the pan‐HER kinase inhibitor neratinib included nine patients with *HER2*‐mutated BTC; the objective response rate at week 8 was 22.2% and clinical benefit (stable disease or partial response lasting at least 24 weeks) rate was 33.3%.^41^

## 4 TYROSINE RECEPTOR KINASE (*TRK*) FUSION GENE

*NTRK1*, *NTRK2*, and NTRK3 encode the neurotropic receptor tyrosine kinases, TRKA, TRKB, and TRKC.^42^ Overexpression of chimeric proteins due to TRK fusions result in dynamic ligand‐independent downstream signaling.^42^ Molecular biology experiments and earlier clinical information propose that these fusions result in oncogene dependency irrespective of the tissue's source, suggesting that it may act as a risk factor in approximately 1% of all the solid tumors.^43^, ^44^, ^45^, ^46^, ^47^, ^48^ Ross et al^49^ reported that *NTRK* fusion‐positive rate was 5.6% in patients with intrahepatic cholangiocarcinoma (Table 1).

Based on scientific evidence, the entire response rate of larotrectinib in *TRK* fusion‐positive tumor types was nearly 80% (95% CI, 67‐90), irrespective of the tumor type.^42^ Studies show that specific mutations can be created to cope with the acquired mutations in the kinase domain; for instance, LOXO‐195 is presently being assessed in adults and children in a phase I‐II study.^42^ Larotrectinib‐related adverse events that resulted in dose reductions were unusual in this study; in a study of 55 patients with TRK fusion‐positive cancer, therapy was not halted for any of the patients due to drug‐related unwanted effects.^42^ Larotrectinib had noticeable and durable anti‐tumor action in patients with *TRK* fusion‐positive cancers, irrespective of the chronological age of the affected person or tumor variety. Long‐lasting responses were noticed irrespective of patient age, tumor tissue, and the position of fusion.^42^ Long‐term management with larotrectinib is possible for patients with minimal side‐effects.^42^ Nevertheless, another study regarding extended follow‐up of a larger patient cohort indicated that experience may offer additional comprehension of the safety profile of this agent.

## 5 FIBROBLAST GROWTH FACTOR RECEPTOR (*FGFR)* FUSION GENE

In a recent study on whole exome and transcriptome sequencing, *FGFR2* fusions were recognized in two of the four cholangiocarcinomas sequenced (50%).^50^ *FGFR2‐BICC1* fusion was recognized in both cases.^50^ A NGS‐based diagnostic assay showed that two‐thirds of the intrahepatic cholangiocarcinoma patients harbored possibly achievable gene changes, which can be used for developing customized therapies and selecting patient‐specific therapies in clinical trials.^50^ Considering the constrained treatment plans, inadequate prognosis in intrahepatic cholangiocarcinoma patients, and the diversity of workable variations mentioned in this study, extensive genomic profiling can promote innovation of treatment models and assist in rectifying an unsatisfactory clinical requirement. In a phase II study of BGJ398 in 61 patients with *FGFR*‐altered advanced cholangiocarcinoma, the overall response rate was 14.8% (18.8% *FGFR2* fusions only), disease control rate was 75.4% (83.3% *FGFR2* fusions only), and estimated median progression‐free survival was 5.8 months (95% CI, 4.3‐7.6 months).^51^

## 6 ISOCITRATE DEHYDROGENASE (*IDH)* ALTERATIONS

Mutations in the genes encoding isocitrate dehydrogenase (*IDH1* and *IDH2*) are observed more regularly in noninfectious cholangiocarcinomas.^52^ *IDH1* and *IDH2* mutations were also identified (19%) in the Johns Hopkins group.^52^ These mutations were grouped in formerly recognized hot spots (codons 132 and 172) and were related to poor prognosis.^53^, ^54^ These variations in analysis could be because of differences in sample size and in the basic features of these two studies.^53^, ^54^ A Chinese study reported only five (4.9%) patients with intrahepatic cholangiocarcinoma who harbored *IDH1* mutations.^55^

## 7 MICROSATELLITE INSTABILITY‐HIGH (MSI‐H) & HYPERMUTATION

Mutational load has been shown to be elevated in tumors that can be effectively eradicated using immunotherapies, for instance, in melanoma and lung cancer.^56^ For example, therapy using checkpoint inhibitors in tumors with mismatch repair deficiency was shown to be effective in a phase II study, attaining approximately 40% of the target results.^57^ Mutational load is high in BTCs.^57^ Le et al^58^ assessed the efficiency of PD‐1 blockade in patients with advanced mismatch repair‐deficient cancers, including 12 different tumor types; objective radiographic response rate was observed in 53% of patients, and complete response rate was observed for 21% of patients. Detection of tumor hypermutation in cancer is expected to not only predict the clinical benefit of immune checkpoint inhibitor treatment, but also provide better surgical strategies for patients with hypermutated tumors.^59^ Nakamura et al^26^ reported that hypermutated cases, where the high mutation load created abundant tumor‐specific neoantigens, were significantly enriched in immune checkpoint genes (cluster 4); they also evaluated the expression of nine targetable immunosuppressive immune checkpoint molecules, including PD‐L1 (CD274), and the expression of these molecules was significantly higher in cluster 4 than in other clusters. In total, 45.2% of cases showed increase in the expression of immune checkpoint molecules, including those associated with favorable clinical response to treatment with an anti‐PD‐L1 antibody.^26^

The outcomes differed among case series; high‐level MSI has been revealed in 5% of gallbladder carcinoma,^60^ 5%‐13% of extrahepatic cholangiocarcinoma,^60^, ^61^ and up to 10% of intrahepatic cholangiocarcinoma.^60^ Mismatch repair (h*MLH1* and h*MSH2* negativity) was observed in 51.3% and 59% of cases of gallbladder carcinoma and 57.1% and 65.7% of cases of extrahepatic cholangiocarcinoma, respectively.^62^ In addition, O (6)‐methylguanine‐ DNA methyltransferase (MGMT) methylation was acknowledged in 59% of gallbladder carcinoma and 60% of extrahepatic cholangiocarcinoma cases.^62^ Both MGMT methylation and mismatch repair status were related to poor prognosis in gallbladder carcinoma and extrahepatic cholangiocarcinoma.^63^

## 8 GERMLINE VARIANTS IN BILIARY TRACT CANCER

The genetic attributes of BTC are not completely understood, and its molecular profiles are heterogeneous. Large sample sizes are required for extensive evaluation of the molecular basis of BTC. Individuals with germline mutations in breast cancer gene 2 (*BRCA2*) are at high risk of BTC as well as of pancreatic cancer.^64^ In a recent study involving 412 BTC samples from Japanese and Italian populations, 32 frequently mutated genes, including a novel deletion of *MUC17* at 7q22.1, were recognized, some of which adversely affected clinical prognosis.^65^ The other significantly and commonly mutated genes included *TP53*, *KRAS*, *SMAD4*, *NF1*, *ARID1A*, *PBRM1*, and *ATR*, some of which negatively affected patient prognosis.^65^ Notably, they observed that at least 11% of BTC cases had deleterious germline mutations in cancer‐predisposing genes.^65^ Zou et al^55^ revealed that *TP53* mutations are more likely to be HBsAg‐seropositive, whereas *KRAS* mutations are nearly exclusively found in HBsAg‐seronegative patients with intrahepatic cholangiocarcinoma.

## 9 TARGETED THERAPY OF INTRAHEPATIC CHOLANGIOCARCINOMA

Currently, authorized medications for treating intrahepatic cholangiocarcinoma are lacking. The current application of next‐generation DNA sequencing expertise in medical practice has allowed oncologists to customize treatment choices for patients in accordance with the inherited alterations triggering the disease. The initiatives for determining targetable genomic improvements using NGS are resulting in the recognition of new and continual gene fusions in various cancers. Mutations in the critical factors of the RAS and PI3K signaling pathways are targeted for treating patients with intrahepatic cholangiocarcinoma.^49^ A study reported several changes in *FGFR2*, such as three‐gene fusions, while another report identified *FGFR2* fusions in primary hepatic cholangiocarcinoma.^49^ In particular, the most common variations were within *ARID1A* (36%), *IDH1/2* (36%), and *TP53* (36%), in addition to the amplification of *MCL1* (21%).^49^ Nearly 66% of patients within this study harbored genomic changes, which might be associated with targeted treatments, and, therefore, therapy options can be possibly custom‐designed for individual patients.^49^ In the liver biopsy of a 62 year‐old female patient, Ross et al^49^ applied a NGS filtering process of 28 formalin‐fixed paraffin‐embedded (FFPE) samples of intrahepatic cholangiocarcinoma, and identified a new gene fusion, *RABGAP1L‐NTRK1* (3.6%). In addition, a repeated gene fusion of *ETV6* and *NTRK3* (*ETV6‐NTRK3*) has been defined in congenital fibrosarcoma.^66^

## 10 PRECISION MEDICINE AND IMMUNOTHERAPY IN BILIARY TRACT CANCER

Currently, IDH inhibitors for *IDH*‐mutant BTC and molecules targeting *FGFR2* gene fusions are being used for treating BTC. The majority of the outstanding molecular targets that have been analyzed in clinical studies have yielded relatively unsatisfactory outcomes, with inconsistent results and unfavorable trials, indicating that unknown targets/pathways and better methods for understanding the complicated molecular biology of BTC are required.^22^ As with other malignancies, significant reduction in the cost of NGS technological innovations has facilitated additional advanced trials, using various molecular subtypes of the metastasizing cancer that can be associated with specific inhibitors. Acquiring the tumor molecular profiles of patients who are fit to join clinical studies outside the first‐line systemic therapy can offer these patients further encouraging treatment plans. On the other hand, acquiring adequate BTC tissue for such purposes can be challenging, thereby complicating this strategy. Owing to these circumstances, the use of liquid biopsies, for instance, circulating tumor cells, cell‐free DNA, and exosomes, should be optimized to obtain robust and favorable outcomes.

Innate and adoptive immune cells are found in BTCs; this is apparently the phase structure (for macrophages), and the existence of dendritic cells, CD4+ helper T‐lymphocytes, CD8+ cytotoxic T‐lymphocytes, and B‐lymphocytes/plasma cells are associated to be enhanced tactical.^67^ MUC1, a glycoprotein forming a hydrophilic barrier to hydrophobic cytotoxic agents and immune system surveillance, is overexpressed in gallbladder carcinoma (90%), but relatively poorly expressed in cholangiocarcinoma (59%‐77%), and is associated with an advanced stage of the disease and reduced survival. A previous study revealed that MUC1 vaccination failed to produce clinical gains despite eliciting an IgG response.^68^ Shimizu et al vaccinated patients with resected intrahepatic cholangiocarcinoma with autologous tumor lysate‐pulsed dendritic cells plus ex‐vivo‐activated T‐cell transfer (adoptive immunotherapy). The overall survival of these patients was double (31.9 vs 17.4 months, *P* = .022) of that of surgery‐alone patients; this was most marked in patients with prominent skin reactions.^69^

## 11 KRAS‐BRAF‐MEK‐ERK PATHWAY

As with several types of cancer, the RAS‐RAF‐MEK‐ERK signal transduction pathway is often dysregulated in cholangiocarcinoma.^70^ Epidermal growth factor (EGF) and platelet‐derived growth factor (PDGF) trigger a cascade of activation of downstream signaling molecules. Activated RAS triggers phosphorylation and activation of RAF kinase, ultimately causing end phosphorylation of MEK1 and MEK2. Activated MEK phosphorylates ERK1 and ERK2. Phosphorylated ERK (pERK) then dimerizes and translocates to the nucleus,^71^ where it regulates numerous essential cellular functions. Gain‐of‐function *KRAS* mutations occur in 9%‐40% cases of cholangiocarcinoma.^49^, ^72^ *KRAS* mutation has been related to perineural intrusion, advanced stage disease, and inadequate prognosis.^73^ *KRAS* mutations have also been detected in up to 7.8% cases of gallbladder carcinoma.^19^ *BRAF* mutations are rare in gallbladder carcinoma and are mostly detected in intrahepatic cholangiocarcinoma.^74^, ^75^ Irrespective of the high frequency of occurrence of *KRAS* mutations, targeting of this pathway is always complicated. *BRAF* is a proto‐oncogene and an essential component of the RAS‐ RAF‐MEK‐ERK signaling pathway.^17^ New interdisciplinary approaches focusing on various molecules in this specific pathway or trials for identifying better diagnostic methods for cholangiocarcinoma are required.

Aberrant activation of the RAS‐RAF‐MEK‐ERK pathway in patients with BTC indicates its importance in the treatment of BTC. The KRAS (G12C) inhibitor AMG510 drives anti‐tumor activity, and has reached the clinical testing stage in human solid tumors (clinicaltrials.gov identifier NCT03600883). In preclinical analyses, treatment with AMG 510, which is the first identified KRAS (G12C) inhibitor, led to the regression of KRASG12C tumors and improved the anti‐tumor efficacy of chemotherapy and targeted agents.^76^ In clinical trials, AMG 510 demonstrated anti‐tumor activity in the first dosing cohorts and represented a potentially transformative therapy for patients for whom effective treatments are lacking. Preclinical data indicate inhibition of cell growth in BTC models with *KRAS*‐mutated cell lines after treatment with MEK inhibitors.^77^ Several clinical trials showed that the MEK inhibitor was well‐tolerated and showed promising evidence of activity in patients with BTC.^78^, ^79^

## 12 CONCLUSIONS

The treatment options for patients with advanced BTC are improving; owing to intercontinental cooperation toward understanding and treating BTC, the latter can no longer be considered “rare diseases.” Studies have demonstrated that BTC includes subgroups with individually distinct driver mutations, most of which can be targeted with new treatment plans. Systemic treatment plans, such as targeted therapies and immunotherapy for BTC, is improving rapidly. In addition, development of numerous pathway‐targeted therapies, together with modulation of the immune environment, provides assurance to patients with these disorders. For decreasing the incidence of BTC, robust specialized medical advancement, along with basic and translation analysis, is required. Identification of inherited driver mutations and translational research are required for providing a distinct opinion regarding the past, present, and future of BTCs.

## DISCLOSURE

Funding: Authors declare no financial support for this article.

Conflict of Interest: Authors declare no conflicts of interest for this article.

Author Contribution: Conception and design by TW; data collected by TW, MN, YS, PP, and JS; manuscript written by TW, MN, YS, PP, and JS.
